# Pancreatic carcinosarcoma with rare long-term survival: Case report and review of the literature

**DOI:** 10.1097/MD.0000000000005966

**Published:** 2017-01-27

**Authors:** Zhe Jia, Ke Zhang, RongHai Huang, XinGang Zhou, Li Jiang

**Affiliations:** a Department of General Surgery; b Department of Pathology, Beijing Ditan Hospital, Capital Medical University, Chaoyang District, Beijing, P.R. China.

**Keywords:** adenocarcinoma, carcinosarcoma, gemcitabine, osteosarcoma, pancreas, Whipple procedure

## Abstract

**Patient concerns:**

We report a rare case of pancreatic carcinosarcoma involving a 44-year-old woman. The patient complained of discomfort associated with the upper abdomen and jaundice of skin and sclera for 1 week.

**Diagnoses:**

After hospitalization, relevant examinations were completed. The disease was diagnosed as carcinoma of the pancreatic head.

**Interventions:**

Whipple procedure was conducted in May 2013. Intraoperative exploration indicated 2 components of the tumor: a fish-shaped gray matter and a hard structure similar to cancellous bone. Histopathological examination showed adenocarcinoma and osteosarcoma. After surgery, the patient received 8 cycles of chemotherapy with gemcitabine and raltitrexed.

**Outcomes:**

Previous studies indicated poor prognosis for pancreatic carcinosarcoma. However, our patient survived for 31 months with no recurrence till date.

**Lessons subsections:**

Coexistence of pancreatic adenocarcinoma and osteosarcoma is very rare. Our case was also an exception in manifesting longer survival than expected.

## 1 Introduction

Carcinosarcoma is a rare malignant tumor with coexisting carcinoma and sarcoma. Approximately, 20 cases have been reported in the literature. The diagnosis of this disease is usually based on immunohistochemical examination of resected specimens.^[1]^ Previous reports indicated poor prognosis. Gelos et al^[2]^ confirmed that the average postoperative survival time was 6 months. The case was unique in that the pancreatic carcinosarcoma comprised 2 independent components: ductal adenocarcinoma and osteosarcoma. The patient survived for a long time without recurrence. We have, therefore, discussed this case, and also reviewed the relevant literature. The patient signed an informed consent and agreed to participate in this study.

## 2 Case report

**Figure 1:**
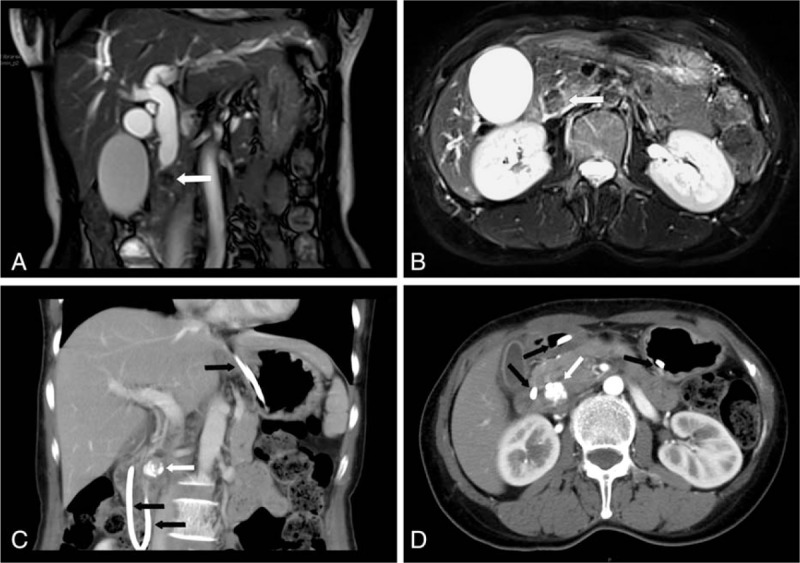
Abdominal MRI (A, B): nodular low-signal shadow (white arrow) was seen at the lower segment of common bile duct. Abdominal contrast-enhanced CT (C, D): nodular high-intensity shadow (white arrow) was observed in the upper duodenum. (Black arrow indicating nasobiliary drainage). CT = computed tomography, MRI = magnetic resonance imaging.

The patient was a 44-year-old woman. She complained of discomfort in the upper abdomen lasting over 1 week, accompanied by progressively exacerbated jaundice of the skin and sclera. The color of her urine was dark, and the stool was pale. Color Doppler ultrasound examination at a community hospital indicated space-occupying lesions in the pancreas and common bile duct dilation. The patient underwent appendectomy 4 years ago and epiglottic cystectomy 2 years ago. The patient has no history of smoking or alcoholism. The patient's sister underwent local excision of colonic carcinoid by electronic colonoscopy in October 2014. No explicit familial history of cancer was found. Physical examination at our hospital did not indicate any abnormalities. Laboratory examination showed the following results: alanine aminotransferase, 482.1 U/L (0∼40 U/L); aspartate aminotransferase, 345.6 U/L (13∼35 U/L); total bilirubin, 184.2 μmol/L (3.4∼17.1 μmol/L); direct bilirubin, 149.6 μmol/L (0∼6.8 μmol/L); and CA-199 >1200 kU/L (<37 kU/L). Amylase and lipase levels were within the normal range. Abdominal color Doppler ultrasound indicated dilation of the intra- and extra-hepatic bile ducts, and the presence of a 2.9 × 1.6 cm hypoechoic area in the lower segment of common bile duct. Abdominal magnetic resonance imaging (MRI) and Magnetic Resonance Cholangiopancreatography (MRCP) indicated nodular abnormal signals at the head of pancreas. Mild signal enhancement suggesting intra- and extra-hepatic bile duct dilatation, and narrowing of distal common bile duct, was observed. Lesions of the head of pancreas were found (Fig. 1A, B). Endoscopic retrograde cholangiopancreatography (ERCP) was conducted before surgery, which revealed normal duodenal papilla. Cholangiography showed stenosis at the lower segment of common bile duct, and dilation of the upper-middle segment of common and intrahepatic bile ducts. Endoscopic nasobiliary drainage (ENBD) was used to relieve the symptoms of jaundice. Abdominal contrast-enhanced computed tomography (CT) was conducted 1 week later, and the results showed a nodular high-density image at the uncinate process of the head of pancreas. Enhanced scan revealed no obvious enhancement, and the diameter was about 1.2 cm (Fig. 1C, D).

**Figure 2:**
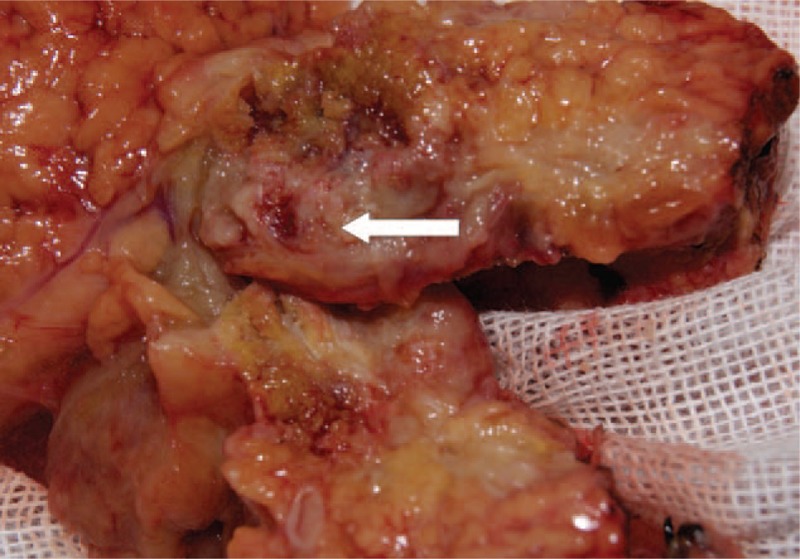
Postoperative specimen: spherical tumor at the head of pancreas, measuring 3 cm in diameter. It was composed of a fish-shaped gray matter and a hard structure resembling spongy bone in close proximity.

**Figure 3:**
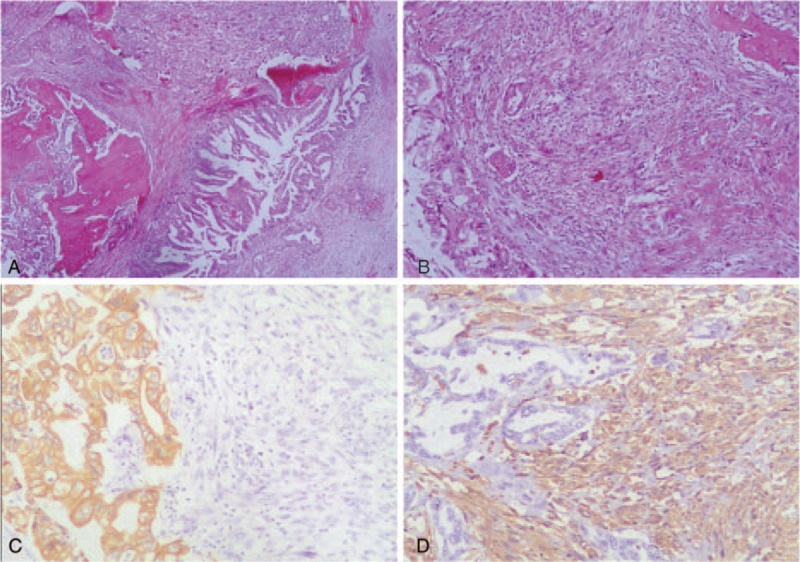
(A) Hematoxylin-Eosin (HE) staining ×40: adenocarcinoma (right lower part) with dysmorphic spindle cell proliferation (right upper part) and neoplastic osteogenesis (left lower part). (B) HE ×100: adenocarcinoma composed of moderately differentiated dysmorphic and proliferating pancreatic duct-like structure. Spindle cells in the osteosarcoma region were obviously dysmorphic. Pathological mitotic and neoplastic osteogenesis was observed. (C) Immunohistochemical (IHC) staining: cytokeratin7-expression in adenocarcinoma, but not in osteosarcoma (Envision method, ×200). (D) IHC: vimentin-expression in osteosarcoma, but not in adenocarcinoma (Envision method, ×200).

Comprehensive analysis suggested the presence of space-occupying lesion at the head of pancreas, not excluding the possibility of malignant tumor. Therefore, we conducted Whipple procedure in May 2013. Postoperative pathological findings indicated a spherical tumor at the uncinate process of the pancreatic head, with a diameter of 3 cm. It was composed of fish-shaped gray-matter and a hard structure similar to spongy bone. These two components were in close proximity (Fig. 2). The pancreatic tumor was confirmed as carcinosarcoma consisting of moderately differentiated adenocarcinoma and heterologous mesenchymal osteosarcoma (Fig. 3A, B). The tumor invaded the fat tissues surrounding the head of pancreas. Lymph node metastasis surrounding the head of pancreas was seen (3/4). The tumor stage was determined as T_3_N_1_M_0_. Biphasic histological differentiation was observed, suggesting moderate-to-mild adenocarcinoma. The tumor tested positive for the epithelial markercytokeratin7 and mesenchymal marker vimentin in immunohistochemical analysis. The tumor originated in the pancreatic duct and contained malignant spindle cells (Fig. 3C), accompanied by mononuclear and giant polymorphonuclear cells. Osteoid and bony tissues of mesenchymal origin were found (Fig. 3D). The two components invaded the surrounding pancreatic tissues, and only the adenocarcinoma spread to the lymph nodes.

The patient was discharged 2 weeks after surgery. The T-tube was removed in the 4th week after surgery. The patient received 8 cycles of adjuvant chemotherapy of gemcitabine 1000 mg/m^2^ (D1, D8) combined with raltitrexed 3 mg/m^2^ (D1) continuously. Regular review was conducted during chemotherapy. With the exception of Carbohydrate Antigen 19-9 (CA19-9), which fluctuated between 50.1 and 425.1 kU/L, all the other laboratory and imaging findings indicated the absence of tumor recurrence or distant metastasis. At 21 months postsurgery, CA-199 decreased to normal level. Follow-up at 31 months postoperatively indicated continued absence of tumor recurrence.

## 3 Discussion

Pancreatic carcinosarcoma is an extremely rare tumor with coexisting carcinoma and sarcoma. Currently, more than 20 case reports are available. We summarized and analyzed 19 cases (Table 1), with relatively complete data.^[1–19]^ We found that pancreatic carcinosarcoma was common in elderly, and rare in young adults (61.9 ± 14.5 years old). Lee et al^[3]^ reported a 24-year-old patient as an exceptional case. The percentage of women was slightly higher at 57.9%. The possibility of tumor incidence at the head of pancreas was twice that of pancreatic body and tail. Large differences in tumor size (2.2–30 cm) were seen. The disease was not specific before operation. Most patients manifested abdominal pain or obstructive jaundice at an early stage. The disease was usually detected during physical examination^[4]^ or while treating other diseases such as anemia,^[2]^ deep vein thrombosis of lower limb,^[5]^ or glucose abnormality.^[6]^ The levels of tumor marker CA-199 were obviously increased in most patients. No typical characteristics of carcinosarcoma were seen during imaging.^[7]^ In our patient, osteosarcoma was misdiagnosed as a high-density stone or calcified lymph node based on imaging. Thus, preoperative diagnosis of this disease is difficult, and definitive diagnosis depended on postoperative tumor morphology and pathological evaluation.

**Table 1:**
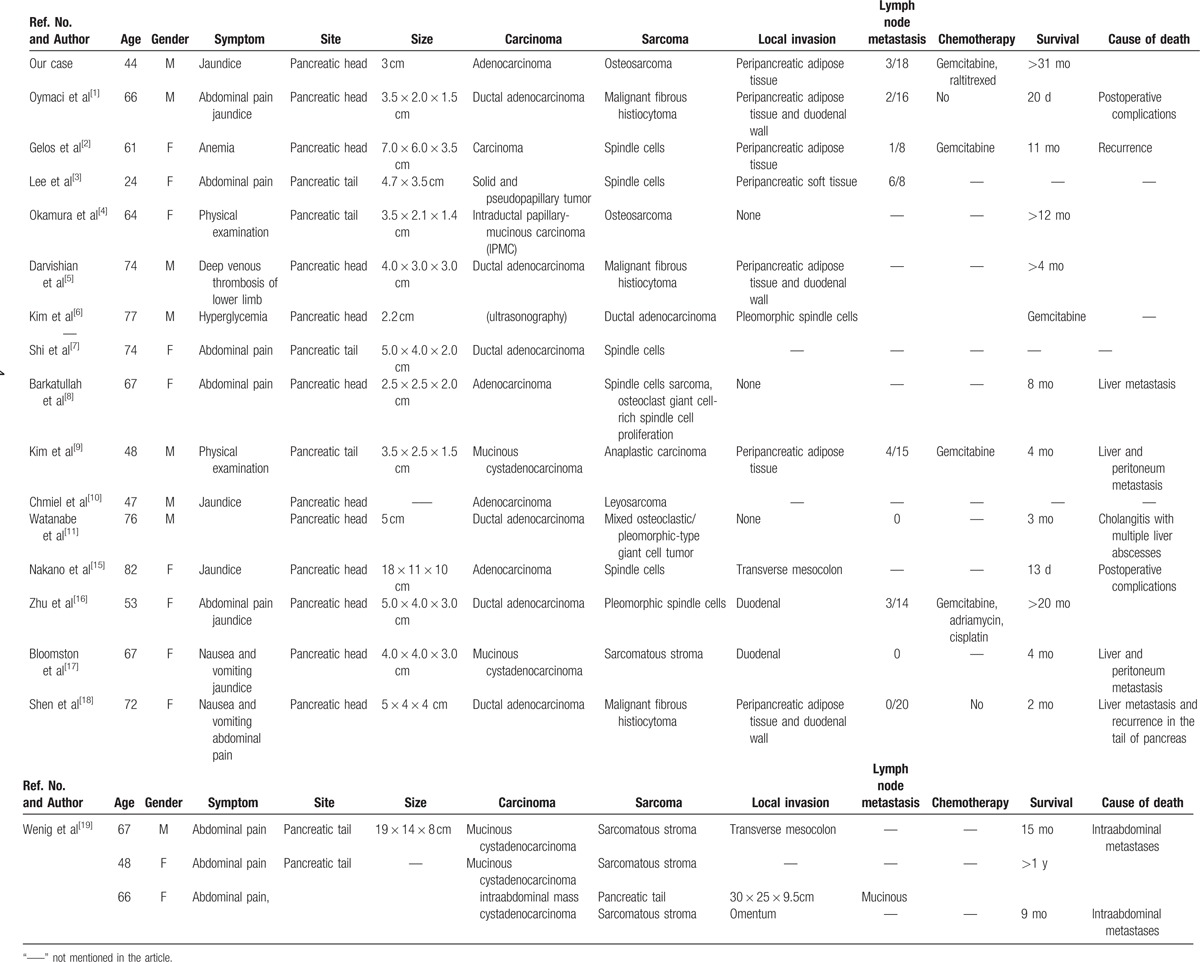
A detailed analysis of 19 cases.

Carcinosarcoma comprises coexisting carcinoma and sarcoma. Based on pathological diagnosis, 2 or more malignant components are required for carcinoma diagnosis. The carcinoma tests positive for epithelial tissue markers cytokeratin (such as cytokeratins 7, 8, 18, 19, and pan-cytokeratin AE1/AE3), and the sarcoma component is positive for mesenchymal tissue marker vimentin.^[2]^ Most pancreatic carcinosarcomas are composed of carcinoma and sarcoma components, one each. However, the pancreatic carcinosarcoma reported by Barkatullah et al^[8]^ consisted of 3 different components: adenocarcinoma, high-grade undifferentiated spindle cell sarcoma, and osteoclast giant cell-rich spindle cell proliferation. Most of the cases reviewed by us were ductal adenocarcinoma, mucinous cystadenocarcinoma,^[9]^ and intraductal papillary mucinous carcinoma.^[6]^ Due to the low degree of differentiation, sarcoma in most cases consists of polymorphic spindle cells. However, leiomyosarcoma,^[10]^ osteosarcoma,^[4]^ and osteoclast giant cell tumor^[11]^ were predominantly seen in other cases.

The mechanism of carcinosarcoma is unclear. Three possible mechanisms have been proposed:

(1) Single early-stage carcinoma, and partial transformation into sarcoma^[12]^;
(2) Tumors of different origin in close proximity, during growth, but without mutual integration^[13]^; and
(3) Single stem cell-differentiation into epithelial and mesenchymal cells.^[14]^

However, recent evidence suggests that monoclonal origin from a single stem cell was likely. Kim et al^[9]^ detected point mutation at the second exon of codon 12 on *KRAS* gene. Additionally, Nakano et al^[15]^ showed that mutations not only occurred at the second exon of codon 12 but also at codon 34. We are currently investigating the homology between carcinoma and sarcoma in this patient.

Treatment options are similar to those of pancreatic carcinoma. Radical resection is the best option for patients contraindicated for surgery. Systemic chemotherapy is indicated for patients with distant metastasis or contraindication to surgery. Chemotherapy is an important secondary option for pancreatic carcinoma. However, there are no relevant standard chemotherapies available for the different types of tumors with different sensitivity. Therefore, chemotherapy for pancreatic carcinosarcoma is very difficult. We administered 8 cycles of chemotherapy including gemcitabine combined with raltitrexed. Most of the cases reviewed in the literature, were treated with gemcitabine alone, while a few received gemcitabine combined with doxorubicin, cisplatin, and other drugs.^[16]^ Due to the extremely limited sample size, no differences in treatment efficacy were observed with various postoperative adjuvant chemotherapies.

Malignancy of pancreatic carcinosarcoma is usually high. Previous studies indicated invasion of peripancreatic adipose tissue^[2,3,4,5,9,18]^ and duodenal wall^[5,16,17,18]^ in most cases, or metastasis of surrounding lymph nodes^[2,3,5,9,16]^ and other organs such as liver,^[6,15]^ resulting in poor prognosis. Gelos et al^[2]^ found that the average postoperative survival time was 6 months. We found a median survival time of 9 months using Kaplan–Meier method. The primary cause of mortality was severe postoperative complications^[1,11,15]^ or carcinoma peritoneum and liver metastasis.^[2,8,9,17,18,19]^ Zhu et al^[16]^ reported a survival period of 20 months in a single patient. However, in our case, although pathological results indicated tumor invasion of peripancreatic adipose tissue and lymph nodes at the head of the pancreas, and abnormal fluctuations in CA-199 postsurgery, the patient survived for 31 months. Based on the follow-up results, our patient showed no recurrence.

## 4 Conclusion

Patients with pancreatic carcinosarcoma manifest non-specific preoperative symptoms and signs, and definitive diagnosis is based on postoperative pathological findings. Radical resection is a reasonable option. However, the choice of treatment should be based on the pathology of carcinosarcoma and is highly individualized. Although our patient survived long after surgery, previous reports indicate poor prognosis due to low differentiation, vascular thrombosis and nerve invasion, and distant metastasis to other tissues and organs at the time of diagnosis. In our patient, tumor invasion to the lymph node surrounding the head of pancreas (the first station lymph node) occurred without any distant metastasis. R_0_ resection of the tumor (microscopically negative surgical margins), complete cleaning of lymph nodes, as well as postoperative adjuvant chemotherapy with gemcitabine combined with raltitrexed, may result in better prognosis.
